# Supplementary material for: Mothers’ satisfaction with health extension services and the associated factors in Gamo Goffa zone, Southern Ethiopia
Source: PLoS One. 2020 May 7;15(5):e0232908. doi: 10.1371/journal.pone.0232908 (PMC7205287; doi:10.1371/journal.pone.0232908)
Supplement: S1 File — (DOCX) [file pone.0232908.s001.docx]

**ENGLISH VERSION QUESTIONNAIRE**

**Questionnaire to assess mother’s satisfaction and associated factors with health extension services**

**Information Sheet**

Good morning/ Good afternoon. My name is__________________, I am working as data collector in this study, which assesses level of mother’s satisfaction and associated factors with health extension service in Gamo Gofa Zone for an investigator doing his thesis for the partial fulfillment of master’s degree in public health at Arba Minch University. You are kindly requested to participate in this study and provide the information required from you. Your participation in this study is completely on voluntary bases and you have the right to refuse, to take part or to interrupt at any time. But your participation will give us quite useful information to take important strategies to the mother’s satisfaction. There are no incentives to participate in this study. We would like to assure you, your name will not be mentioned in anywhere. The information that you will give us will be kept confidential and only used for the research purpose. The questionnaire will take 20-25 minutes.

So, are you willing to participate in this study? Yes/No

If the answer is yes, thanks and continue to fill the questionnaire and if the answer is no transfer to the next respondent

Questionnaire code number _______________

Name of the data collector________________ Date of interview _________Signature ________

Name of the supervisor ___________________ Date of checking _______Signature _________

1. **Socio-demographic factors**
   1. What is your age now? __________
   2. What is your religion ? Orthodox Protestant Muslim Catholic Others/Specify
   3. Which Ethnic group do you belong to? Gamo Goffa Wolayta Amhara Other/specify
   4. What is your marital status? Married Single Divorced Separated Widowed
   5. What is your educational status Can’t read and write Only read and write Primary level Secondary level College/university
   6. What is your occupation? House wife Government Employee Merchant Other/specify______
   7. Would you tell me the average monthly income of your family __________
   8. How many of you are living in your household __________
2. **Respondents experience and interaction with health extension workers**
   1. Have you heard about health extension program (HEP)? Yes No

Yes No Yes No

- 1. If your answer for Q 2.1 is yes, where did you get the information about the HEP? From neighbor From mass media From the health extension workers From the health center Others, specify_____

Yes No Yes No

Yes No Yes No

Yes No Yes No

Yes No Yes No

Yes No Yes No

- 1. Have you ever visited HP during the last one year? Yes No

Yes No Yes No

Yes No Yes No

- 1. If yes, how many times did you visit in the last one year? 1 times 2 times 3 times greater than or equal to 4 times

Yes No Yes No

Yes No Yes No

Yes No Yes No

Yes No Yes No

- 1. How long does it take on foot to you to reach the nearby HP (in minute) _____________
  2. How do you rate the availability of HEWs on job at health post? Always Occasional Rarely
- Yes No Yes No

Yes No Yes No

Yes No Yes No

- 1. Have you ever returned home due to HP being closed? Yes No
- Yes No Yes No
- Yes No Yes No
  1. Did you receive a service from health extension workers in the HP? Yes No

Yes No Yes No

Yes No Yes No

| Services | Tick (🗸) if delivered |
| --- | --- |
| Counselling on nutrition |  |
| FP |  |
| ANC |  |
| Immunization |  |
| Delivery |  |
| Breastfeeding |  |
| Environmental sanitation |  |
| Excreta disposal |  |
| Solid and liquid waste disposal |  |
| Food supply and safety measures |  |
| Water supply and safety measures |  |
| Personal hygiene |  |
| Insect and rodent control |  |
| First aid |  |
| HIV/AIDS, Other STDs |  |
| Malaria |  |
| TB |  |

- 1. If your answer for Q 2.8 is yes; what service(s) have you got from HEW?
  2. Did HEWs visit your home during the last one year? Yes No

Yes No Yes No

Yes No Yes No

- 1. If your answer for Q 2.10 is yes, how frequently did they visit your home during the last one year? one times two times three times >four times

Yes No Yes No

Yes No Yes No

Yes No Yes No

Yes No Yes No

- 1. Do the HEWs involve your husband? Yes No

Yes No Yes No

Yes No Yes No

- 1. Did you participate in planning of health extension activities in the last year?

Yes No

Yes No Yes No

Yes No Yes No

- 1. Do you participate in the activity of health development army?

Yes No

Yes No Yes No

Yes No Yes No

- 1. Are you recognized as a model family? Yes No

Yes No Yes No

Yes No Yes No

- 1. If your answer for Q 2.15 is No, do you Know the model families in the HEP in your Kebele? Yes No

Yes No Yes No

Yes No Yes No

- 1. Where do you think HEWs live? HP Town With family Others/Specify ________________

Yes No Yes No

Yes No Yes No

Yes No Yes No

Yes No Yes No

- 1. Do you think that the service provided by HEWs is enough? Yes No

Yes No Yes No

Yes No Yes No

1. Mothers’ satisfaction with the service delivered by Health Extension Program (HEWs). Please Tick (🗸) the appropriate answer in the box.

Note: 5= Very satisfied, 4 = Satisfied, 3 = Neutral, 2= Dissatisfied, 1 = Very Dissatisfied

| S.No | Questions | 1 | 2 | 3 | 4 | 5 |
| --- | --- | --- | --- | --- | --- | --- |
| **Convenience** | | |  |  |  |  |
| 3.1 | Simplicity and trouble free of Service System Delivered by HEWS. |  |  |  |  |  |
| 3.2 | Availability of needed instruments like BP apparatus, thermometer, others |  |  |  |  |  |
| 3.3 | The practice of HEWs to refer for consultation when needed. |  |  |  |  |  |
| **Courtesy (a polite speech or action)** | | |  |  |  |  |
| 3.4 | Friendliness of HEWs and courteous manner of HEWs |  |  |  |  |  |
| 3.5 | The attentiveness of HEW while answering your question |  |  |  |  |  |
| 3.6 | Provide appropriate time for examination and counselling |  |  |  |  |  |
| 3.7 | Maintain privacy appropriately before doing any procedure |  |  |  |  |  |
| **Quality of service provision:** Please indicate your level of satisfaction | | |  |  |  |  |
| 3.8 | How do you generally perceive your satisfaction to the HE services? |  |  |  |  |  |
| 3.9 | Was there a variety of services that you wanted? |  |  |  |  |  |
| 3.10 | How would you rate the quality of service you have received from HEW? |  |  |  |  |  |
| 3.11 | To what extent has HES met your needs? |  |  |  |  |  |
| 3.12 | Was the information you wanted to get available and accessible from the HE service? |  |  |  |  |  |
| 3.13 | Have the services you received helped you to deal more effectively with your problems? |  |  |  |  |  |
| 3.14 | If a friend were in need of similar help, would you recommend HES to her? |  |  |  |  |  |
| 3.15 | How satisfied were you with the specific services during home visit by HEWs? |  |  |  |  |  |
| 3.16 | How do you rate your happiness with the HEWs follow-up service in the health posts? |  |  |  |  |  |
