# Supplementary material for: Mothers’ satisfaction with health extension services and the associated factors in Gamo Goffa zone, Southern Ethiopia
Source: PLoS One. 2020 May 7;15(5):e0232908. doi: 10.1371/journal.pone.0232908 (PMC7205287; doi:10.1371/journal.pone.0232908)
Supplement: S2 File — (DOCX) [file pone.0232908.s002.docx]

**Gamotho version questionnaire**

Enoteththa oysha mara (Consent Form)

**Gelo:** Ha oysha shishiya na suntha, oosowa, de7osonne ooso gisha eranawu danda7iyoge niyo maata.

**Pilgeta kara:** Gamo Goffa zonnen Dugeha Thopiyan Xeena extensione programe imettizayssa go7an aayeta allo dethanne digiya gassota

**Pilgiya assa sunthay:** Mark Mergiya

**Osisiya ketsatsa:** Arbaminch Universitya, xallenne payateththa Science College, assa payateththa departimenthe, Gamo Gofa Zonne payateththa ooso ketha

**Pilgetayo huuphphe qofay:** ha pilgety Gamo Goffa zonnen Dugeha Thopiyan Xeena extensione programe imettizayssa go7an aayeta allo dethanne digiya gassota shakidi eranawu maddees.

**Kallo oge:** issitho ha oyshas ne enoteththa koshshees. Ne enoteththy de7ikko guuththa oysha oychchays. Ta nena oychchanayika issi 20 daqiqa.

**Metto/itathaththa:** ta nenna ha pilgeththa baggara oychchiyo oyshay ay methokka ne bolla ehenaga wozinappe erisayiss.

**Go7a:** ha pilgetha muruuta wursetan eranwu neyo mathay de7ees. Giddoppe athin ha pilgetha oysha neni enoteththan zariddo gishsha aybika qanxetiyabi bawaa.

**Xura qotto:** ha pilgethan ne immidda qofan xura gidiyaga eranawu bessees. Ne sunthayka xufen oyketibena.

**Erro woykko akkay giyo matta**: ha pilgethay neni erro gikko xaala osetees. Akkay gikokka onika wolqantenanne hilenna.

**Ha pilgetha oyshas neni ero gayi? A. Ee B. Akkay**

**Hara oyshay deyi? _________________________________________________**

**Gayttiyo assa:** Mark Mergiya, silkya payddoy 0912125987 woykko 093474713

Gamugna Questionnaire

Gamo Goffa zonnen Dugeha Thopiyan Xeena extensione programe imettizayssa go7an aayeta allo dethanne digiya gassota oyshata

Erisiyoba (Information Sheet)

Lo77o aqqadi/lo77o pe7adi. Ta sunthay__________________, tani ha pilgethas allaliya shishays. Ha allalekka Gamo Goffa zonnen Dugeha Tophiyan Xeena extensione programe imettizayssa go7an aayeta allo dethanne digiya gassota shakidi eranawu maddees. Hagekka ba 2^tho^ digree asa payateththan Arbaminche Universityan, xallenne payateththa Science Collegiyan, assa payateththan tamaariyagassa. Ha pilgetha oyshas lo77o zaro immanamala bonchchuwan oychays. Ne immiya qofay kumtara enotethaththaninne ne maatan gidishin ne koyida wodden essanawu danddiyassa. Gido athin ne immiyo qofathi ayetha allo detha gujanas kehippe maddees. Ne sunthay awunkka xaafetibenaga hayika neni eranawu bessees. Ha pilgethan ne immidda qofay xura gidiyagakka eranawu bessees. Ha ne imma qofay pilgetha allaliyas xallala. Ta nena oychchanayika issi 20 daqiqa.

Gididi , Ha pilgetha oyshas neni ero gayi? A. Ee B. Akkay

Zaroy Ee gidiko, galatara oyshsha doma.

Zaroy Akkay gidiko, kaliya oyshanchchawu lama

Maalata paydo ______________

Alalay shikida asa suntha_______________ gaalassa ________ malatha/pirmma ________

Zari xeeliya asa (supervisor) ___________ xelido gaalassa _______ malatha/pirmma ______

Translated Questionnaire (Gamugna)

1. Duussa hanota

Maalata paydo _____ Alalay shikida gaalasa _______ Alalay shikida assa suntan _________

- 1. Laythya __________
  2. Ammano Ortodokkise Misone Isilaame Katolikke Haray De7ikko _____
  3. Beresebe Gamo Goofa Wolaytha Amara Haray De7ikko _____

- 1. So asa bagga Geladus gelabe7ukku Bethe Yadus Higen Birshshadus Hayqqon shaakettides
  2. Timirtte Detha Nabbabeku/xaafuku Nabbabawus/ xaafawus Issantho detha Nam77antho detha kolojje/yunbursha detha
  3. Ootho/ ooso hanota Keethanddo Kawo oosanchcha Zal77anchcha Haray de7ikko_________
  4. Agina demmisha __________
  5. So asaa qooda/payddo __________

1. Zaarizaytta ooso meezenne payyatetha oosanchchatara de7iyo gahetetha/Respondents experience and interaction with health extension workers
   1. Payyatetha ooso bagga (teena extension prograame) siyagadis? Ee Akkay

Yes No Yes No

- 1. Ne zaaroy “Ee” gidikko awappe siyadii?? Shooroppe Tirppa gahetethatappe Payyatetha gxare oosanchchatappe Xeena xaabappe Haray de7ikko _____

Yes No Yes No

Yes No Yes No

Yes No Yes No

Yes No Yes No

Yes No Yes No

- 1. Ha laytha giddon xeena keela ba eray? Ee Akkay

Yes No Yes No

Yes No Yes No

- 1. Ne zaaroy “Ee” gidikko aappuna simmerettadii? Issito Nam77u toho Heedzu toho oyddanne bolla

Yes No Yes No

Yes No Yes No

Yes No Yes No

Yes No Yes No

- 1. Nesoppe xeena keela gaso de7iya ogey ay keena wode ekkii? (apu dakike) ________
  2. Gaxaare xeena-keela oosanchchati ubba wode beettizoo? Ubba wode Aadhi -aadhi Guutha-toho
- Yes No Yes No

Yes No Yes No

Yes No Yes No

- 1. Xeena-keelay gorddetin coo go7a demmontta ne soo simmida wodey de7ii? Ee Akkay
- Yes No Yes No
- Yes No Yes No
  1. Gaxare xeena-keela oosanchchatappe neni go7a demmida wodey de7ii? Ee Akkay

Yes No Yes No

Yes No Yes No

| Payyatetha oosota | Ne go7ettidayssa bolla hano (🗸) malata |
| --- | --- |
| Go7ata/ Haggazo (Nutrition) |  |
| Yelo halchcho |  |
| Yelanappe sinthe go7a (ANC) |  |
| Kitibate |  |
| Yeliso (Delivery) |  |
| Dantho (Breastfeeding) |  |
| Keetha giddo geeshatetha (Health house environment) |  |
| Sheesha keetha (Excreta disposal) |  |
| Buuranne go7ettida haathata (Solid and liquid waste disposal) |  |
| Katha giigisonne naagetethi (Food supply and safety measures) |  |
| Haathanne naagetethi (Water supply and safety measures) |  |
| Buzo geeshatetha (Personal hygiene) |  |
| Qeeri medosatanne eccereta naago ogeta (Insect and rodent control) |  |
| Koyro detha maado (First aid) |  |
| Eeddissenne hara asho gahetethan aadhdhiza harggeta (HIV/AIDS, Other STD) |  |
| Shekkere/uuno (Malaria) |  |
| Goofina xaama/qufe (TB) |  |

Ne zaaroy oysha payddo 2.8 “Ee” gidikko aaze gaxare xeena oosanchchatappe demmido go7ay?

- 1. Ha laythi neso gaaxare xeena oosanchati yii/kaalli eriyona? Ee Akkay
  2. Ne zaaroy oysha payddo 2.10 “Ee “ gidikko aappun toho kaalli xeellidoo? Issito Nam77i toho Heedzu toho Oyddanne bolla

Yes No Yes No

Yes No Yes No

Yes No Yes No

Yes No Yes No

Yes No Yes No

Yes No Yes No

- 1. Gaxare xeena keela oosanchati nenara zorettishe ne addeza/azina/ qofa laamettanas ekkizoo? Ee Akkay

Yes No Yes No

Yes No Yes No

- 1. Halaytha xeena keela ooso qaaxota halchcho giddon ootha eray? Ee Akkay

Yes No Yes No

Yes No Yes No

- 1. Ne xeena ooso qaaxo issino ichchasha cugan maaran qaaxay? Ee Akkay

Yes No Yes No

Yes No Yes No

- 1. Ne xeena ooso qaaxon moodele gida eray? Ee Akkay

Yes No Yes No

Yes No Yes No

- 1. Ne zaaro oysha payddo 2.15 “Akkay” gidikko hara moodele gidita keethata eray? Ee Akkay

Yes No Yes No

Yes No Yes No

- 1. Gaxare xeena oosanchchati awan de7iya eray? Xeena keelan kataman So asatara haray diikko ________________

Yes No Yes No

Yes No Yes No

Yes No Yes No

Yes No Yes No

- 1. Xeena keela oosanchchatan imettiza haggazoy gidee gaada qoppay? Ee Akkay

Yes No Yes No

Yes No Yes No

1. Xeena extensione programe imettizayssa go7an aayeta allo bagga

Hayyana! (🗸) Ha malata saaxine giddon woththite

Qofiso; 5 = keeha alladis 4= alladis 3 = qofay ba 2= allarkke1= keehippe allarkke

| Mp | Oyshata | 1 | 2 | 3 | 4 | 5 |
| --- | --- | --- | --- | --- | --- | --- |
| Giigateth | | |  |  |  |  |
| 3.1 | Gaxare xeenati suurenne metoy baydda go7a immettes |  |  |  |  |  |
| 3.2 | Oosos maaddiza miishshati leemisos suutha sugetha geedariza miishshati, terimometiretinne harati de7oosona |  |  |  |  |  |
| 3.3 | Gaxare xeenati oychikko oysha bolla zore immettes |  |  |  |  |  |
| Bonchchon haasaya go7etha | | |  |  |  |  |
| 3.4 | Gaxare xeena extensione oosanchchati inttenara diza laggetethanne bonchcho hanota |  |  |  |  |  |
| 3.5 | Gaxare xeena extensione oosanchchati intte oyshata maaran zaaro hanota |  |  |  |  |  |
| 3.6 | Gaxare xeena extensione oosanchchati metota bolla paacanas wode imeettes |  |  |  |  |  |
| 3.7 | Gaxare xeena extensione oosanchchati aykko miishsha oosappe sinthe xuura naageettes |  |  |  |  |  |
| Kuussa naageteth | | |  |  |  |  |
| 3.8 | Gaxare xeena extensione oosanchchati immiza haggazon alladii? Alladis giikko ay keena? |  |  |  |  |  |
| 3.9 | Gaxare xeena extensione oosanchchatappe ne koyiza haggazo demmadii? |  |  |  |  |  |
| 3.10 | Gaxare xeena extensione oosanchchatappe ne demmida haggazo kuussas ay giza detha immay? |  |  |  |  |  |
| 3.11 | Ize immiza go7ay ne koshshazara ay keena gaytii? |  |  |  |  |  |
| 3.12 | Gaxare xeena oosanchchati asataso kaali-xeelo detha wostta immay? |  |  |  |  |  |
| 3.13 | Gaxare xeena extensione oosanchchatappe ne demmida haggazoy ay keena ne metotara suuregaytides gaada qoppay? |  |  |  |  |  |
| 3.14 | Ne mala metora dizayti gaxare xeena oosanchchatappe maado demmana mala zoradii? |  |  |  |  |  |
| 3.15 | Gaxare xeena extensione oosanchchati ne meto bolla paacidayssa ay keena ufayettadii? |  |  |  |  |  |
| 3.16 | Gaxare xeena extensione oosanchchati ne kaali xeelizayssan ay keena ufayettadii? |  |  |  |  |  |
