# Supplementary material for: Mothers’ satisfaction with health extension services and the associated factors in Gamo Goffa zone, Southern Ethiopia
Source: PLoS One. 2020 May 7;15(5):e0232908. doi: 10.1371/journal.pone.0232908 (PMC7205287; doi:10.1371/journal.pone.0232908)
Supplement: Attachment — Submitted filename: Response to reviewers.docx [file pone.0232908.s003.docx]

**RESPONSE TO REVIEWS BY EDITOR AND REVIEWERS**

PONE-D-19-34888

Mothers’ satisfaction with health extension services and the associated factors in Gamo Goffa Zone, Southern Ethiopia

**Editor’s comments**

Journal Requirements: When submitting your revision, we need you to address these additional requirements.

1. Please ensure that your manuscript meets PLOS ONE's style requirements, including those for file naming. The PLOS ONE style templates can be found at

http://www.journals.plos.org/plosone/s/file?id=wjVg/PLOSOne_formatting_sample_main_body.pdf and <http://www.journals.plos.org/plosone/s/file?id=ba62/PLOSOne_formatting_sample_title_authors_affiliations.pdf>

Response: We have made the revised manuscript to conform to plosone’s style requirements.

2. Please include additional information regarding the survey or questionnaire used in the study and ensure that you have provided sufficient details that others could replicate the analyses. For instance, if you developed a questionnaire as part of this study and it is not under a copyright more restrictive than CC-BY, please include a copy, in both the original language and English, as Supporting Information. If the original language is written in non-Latin characters, for example Amharic, Chinese, or Korean, please use a file format that ensures these characters are visible.

Response: We have included a copy of the questionnaire used in this study as a “supporting information”.

3. Please state whether you validated the questionnaire prior to testing on study participants. Please provide details regarding the validation group within the methods section.

Response: We really appreciate for your concern on the gap in information with regard to the validity and reliability of the survey instrument. We admit that we have not followed the steps for scale validation in its strict sense. However, we have done things related to face and content validations as the research team discussed on dimensions of satisfaction and the clarity and relevance of the survey questions and reached agreement before testing the questions on the study women. The research team (experts of public health, nursing and midwifery) and a team of local experts working with in the health extension programme were participated in suggesting the components of satisfaction and appropriate wording of questions. Right before data collection, a pre-test was made on 5% (24 mothers) to see the appropriateness of the questions to the mothers in charge and the necessary amendments were made to make the questions suitable to the survey. Moreover, concurrent to the data collection, a reliability analysis was made to check for internal consistency on 162 samples of women (Cronbach’s alpha=0.89). We apologize for not going through the other validation steps which are not commonly done in our country. An additional statement has been provided in the “manuscript with track changes” document.

4. Please amend your current ethics statement to address the following concerns: Please explain why was written consent was not obtained, how you recorded/documented participant consent, and if the ethics committees/IRBs approved this consent procedure.

Response: Thank you for your concern and remarks on this important area. Oral consent was obtained owing to the fact that majority of the rural women in our setup are not able to read and write except few who might have attended a formal/ an informal education or else working in the public/private sector. Before going to obtain oral consent from each participant, a letter of support and approval for undertaking the research in the local setup was obtained from government officials in each kebele. The IRB approved this procedure initially at proposal defense stage for there was no sensitive issue regarding the research task. These statements have been incorporated to the “revised manuscript with track changes” file in the “methods” section.

xxxxxxxxxxxxxxxxxxxxxxxxxxxxxxxxxxxxxxxxxxxxxxxxxxxxxxxxxxxxxxxxxxxxxxxxxxxxxxxxxxxxxxxxxxxxxxxxxxxxxxxxxxxxxxxxxxxxxxxxxxxxxxxxxxxxxxxxxxxxxxxxxxxxxxxxxxxx

**REVIEWERS' COMMENTS**

**Reviewer #1:**

Mothers’ satisfaction with health extension services and the associated factors in Gamo Gofa Zone, Southern Ethiopia

Thank you for inviting me to review the above-mentioned manuscript. Please see my comments as follow:

1.Abstract: Authors should report the odds of mother that had lower satisfaction not mothers with higher satisfaction.

Response: Our categorization of the outcome variable was initially as “satisfied=1” and “dissatisfied=0”. Thus, the success category would be satisfaction and the odds ratio we found from this analysis were >1 (shows a positive association). Therefore, we have reported in favor of higher satisfaction owing to these reasons -the odds ratio is positive and the success variable initially coded as 1 in the logistic regression model was “satisfied” and as 0 was “dissatisfied”.

**Methods**

1.Please mention the name of country along with the region of study.

Response: We have included it in the “methods” section (line 106).

2.Please state the inclusion/exclusion criteria of the study.

Response: We have included it in the “methods” section (L 121-126).

3.The validity and reliability of the questionnaire that authors used in this study did not confirm properly.

Response: We really appreciate your concern on the gap in information with regard to the validity and reliability of the survey instrument. We admit that we have not followed the steps for scale validation in its rigorous sense. However, we have done things related to face validation and content validations as the research team discussed on dimensions of satisfaction and the clarity and relevance of the survey questions and reached agreement before testing the questions on the study women. The research team (experts of public health, nursing and midwifery) and a team of local experts working with in the health extension programme were participated in suggesting the components of satisfaction and appropriate wording of questions. Right before data collection, a pre-test was made on 5% (24 mothers) to see the suitability of the questions to the mothers in charge and the necessary amendments were made to make the questions suitable to the survey. Moreover, concurrent to the data collection, a reliability analysis was made to check for internal consistency on 162 samples of women (Cronbach’s alpha=0.89). Forgive us for not going through the other validation steps which are not commonly done in our country. An additional statement has been provided in the “manuscript with track changes” document.

Results

1.Table 1: for better understanding of economic situation, please exchange the currency of your country to the US Dollar.

Response: We have corrected it to USD in table 1 of the revised manuscript.

2. It was much better, if you have asked about how participants evaluate their economic situation?

For instance-good, poor, moderate. May you please consider this matter in the limitations of the study.

Response: We have included it as a limitation in the “discussion” section of the revised manuscript.

3.Table 1: Please provide the mean and SD for continuous data such as age.

Response: Dear reviewer, the mean and standard deviation of age was already presented in the text under “socio-demographic characteristics of the study participants”.

3. Please provide some midwifery information such as, gravida, para, number of abortions, and the previous mode of delivery…

Response: Dear reviewer, we do not have these data from the outset. Since we did not get any theoretical or literature ground that supports as these factors were associated with satisfaction to a given program, we did not include them as a variable/explanatory variable in this study. Had these variables were included in the model, the probability that they are too much correlated to maternal age or educational status is high. However, if we had the data now, we would have described it here as a “background” characteristic.

5.What authors mean about model family?

Response: This has been defined in the “definitions and measurement” section as – “**Model families:** Households that are trained in some of the components of the HE packages and able to implement these packages and influence their relatives and neighbors to adopt the same practices [27]”. Kindly look to it.

6.Table 4: please put the number and percentages together.

Response: We have revised it accordingly in the revised manuscript.

7.The availability of HEW on job at HP was significant and seems that occasional availability was a source of dissatisfaction, please explain.

Response: This is just related to mother’s satisfaction with the service directly goes with the usual presence or occasional presence or absence of HEWs in the health post. Health extension workers are providers of the service. When women come to get the service they wanted , they will be happy or satisfied when they get the service providers in the respective health posts, most importantly at all times.

**Discussion**

1.Please write the objective of the study at the beginning of the discussion.

Response: Thank you for your directions. We have included our study objectives at the beginning of the “discussion” section.

2.Please mention the limitations of the study at the end of discussion.

Response: Thank you again for your direction here too. We have incorporated the limitations of the study at the end of the discussion.

3.Authors found that only small percentages of mothers are satisfied with the services received from health providers. It might be due to the different instrument that they used in this study and also did not validate it.

Response: We sincerely apologize for the tool validation which should have been done in a rigorous way before we go for data collection. However, we have tried to show what steps we ensue with the tool development (face and content validation), pre-tested the questionnaire on 5% of the sample of women and conducted a reliability analysis on the first 162 mother’s data (Cronbach’s alpha=0.89). We admit the assertion on the small percentage of satisfaction and had already discussed on it in the “discussion” section…...” Though the low satisfaction might be related to the measurement, it implies that satisfaction with the HES is far lower in the study area seen in light of the aspects considered” …. (lines 264-265). We have included a limitation statement in the “discussion” section.

xxxxxxxxxxxxxxxxxxxxxxxxxxxxxxxxxxxxxxxxxxxxxxxxxxxxxxxxxxxxxxxxxxxxxxxxxxxxxxxxxxxxxxxxxxxxxxxxxxxxxxxxxxxxxxxxxxxxxxxxxxxxxxxxxxxxxxxxxxxxxxxxxxxxxxxxxxxx

**Reviewer #2:**

This cross-sectional study which looked at Mothers’ satisfaction with health extension services and the associated factors in Gamo Gofa Zone, Southern Ethiopia is commendable and will add to the already existing body of knowledge on this topic. The title of the study is appropriate, the sample size determination and methodology were systematic. The study investigates important issues related to maternal satisfaction in a low -income country. The subject is relevant for reflections on prevention of maternal deaths in the country.

Over all the manuscript clearly states factors which contribute maternal satisfaction in relation to health extension service provision which in turn may contribute to decrease maternal mortality in the country, I suggest the paper is suitable for publication once after the author come with necessary correction and hope that editors will consider publishing the article.

Response: Thank you very much for your inspiring remarks.

Besides that, I have some points to mention:

1. Some of grammatical errors should be corrected (subject to verb agreement); for example, data is the plural form so it is better to use “were” instead of “was” and see it for “kebeles” as well

Response: Thank you for your corrections. We have made the necessary corrections where appropriate.

2. At abstract: you state compassionate and respectful maternity services; it doesn’t go with your objectives, so the authors should see it again.

Response: Compassionate and respectful care has been mentioned in the “abstract” and the “background” section for its close connection with satisfaction to service and its strategic connection with the current Ethiopian government health sector transformation plan and the SDGs.

3. In addition, the word “Methods” are repeated.

Response: Thank you for your meticulous observation. We have removed one of the “methods” and the “abstract” section.

4. Background is too long and is better to exchange first and second paragraph

Response: We have exchanged the first and the second paragraphs and therefore references number 1-14 have been exchanged accordingly.

5. In method section p12 L 124; you have used design effect; why it is important to use it since you didn’t clearly state weather you have used cluster sampling or not.

Response: Since the study is at zonal level, we have employed multi- stages sampling where the woredas and kebeles were considered as clusters. We have selected some woredas and kebeles to represent the remaining woredas and kebeles because we considered that these woredas and kebeles were externally homogenous with regard to satisfaction to HE services.

6. Under the Results, the labelling of the tables had the title of the whole study repeated under each table. My suggestion would be that Table 1 should be titled: Sociodemographic characteristics of the respondents, Table 2 should be: Mothers’ interaction with health extension services, and so on.

Response: We have written the label in such a way just to conform to the principle in writing titles of tables and figures as it should be self-explanatory- things like where, what, and when should be answered.

7. Again, in table 1under age category it is mentioned as “30-3” see it again.

Response: Thank you very much for your meticulous observation. We have corrected it under “table 1”.

8. When you try to mention study subjects as ‘participants’ in some part and ‘respondents’ I suggest you to use consistent way of writing.

Response: Thank you for your direction here. We have made it to be consistent as “participants”.

9. Table 5 is not clear. There should be a key to explain the single and double Asterix.

Response: Thank you for the correction. We have clarified further about the symbols.

10. In the “discussion section” it was stated as this discrepancy may be due to difference in knowledge of respondents, cultural diversity, satisfaction items and the techniques used to compute overall satisfaction. This study measured satisfaction in a more comprehensive way considering additional aspects of the HES. My biggest concern is the difference in satisfaction items how it can be different as it was Likert scales. Please explain briefly

Response: Thank you for expressing your concern. We found it a confusing expression and removed it from the stated section.

11. It is better to add declaration section in your manuscript.

Response: We did not add a declaration section as part of the manuscript owing to its absence in the “plosone manuscript formatting”. However, almost all the contents known to be part of “declaration” in the other reputable journals are there in the plosone online submission system and we have provided the appropriate response.

xxxxxxxxxxxxxxxxxxxxxxxxxxxxxxxxxxxxxxxxxxxxxxxxxxxxxxxxxxxxxxxxxxxxxxxxxxxxxxxxxxxxxxxxxxxxxxxxxxxxxxxxxxxxxxxxxxxxxxxxxxxxxxxxxxxxxxxxxxxxxxxx

**Reviewer #3:**

Thank you for the opportunity to review this manuscript under title the Mothers’ satisfaction with health extension services and the associated factors in Gamo Gofa Zone, Southern Ethiopia.

Title:

1. It's better to capitalize the first letter in each word in the title.

Response: We have followed the plose one formatting style that the title should be in sentence case.

2. At the beginning of each paragraph leaves a space and begins writing.

Response: Thank you for your recommendation. We have corrected accordingly.

Abstract:

1-In line 34 delete repeated word (method).

Response: Thank you very much for your meticulous observation to improve our work. We have removed the repeated word.

2-In line 41 review the results and style of writing. For example, focuses on the [AOR=4.02 (95% CI 45 2.0, 8.1),

Response: We ask for forgiveness that we could not get the remark here.

3-Not use Abbreviations in the abstract. In line 55. Write done Health Extension Workers.

Response: Thank you for your suggestion. We have made the necessary correction.

Method:

In general, the method needs revision and rearmament of the subtitle. For example:

1-If you agree change the (Study Area and period) to Setting and Duration of the Study. In line 104.

Response: We have corrected accordingly.

2- Clarify the estimated number of total populations in line (108) under title population estimated or Population with estimation of sample size.

Response: The estimated number of women of reproductive age is estimated from the total population by using a conversion factor for Southern Nations, Nationalities and Peoples Region (SNNPR) of Ethiopia. The conversion factor is derived from average percentages of women 15-49 during the most recent census in the region. Sample size is calculated by considering these women of reproductive age as a source population and using the usual assumptions for single population proportion formula.

3-Explain inclusion and exclusion criteria.

Response: Thank you for your recommendation. We have included the inclusion and exclusion criteria to this study.

Results:

1-Revision all tables.

Response: Thank you for your recommendations. We have revised the tables so that it can conform to the plose one table formatting.

2-Table number 2 not clear. Unifying the form of writing numbers and the symbol %.

Response: Thank you for your recommendation. We have improved table 2 according to your suggestion.

xxxxxxxxxxxxxxxxxxxxxxxxxxxxxxxxxxxxxxxxxxxxxxxxxxxxxxxxxxxxxxxxxxxxxxxxxxxxxxxxxxxxxxxxxxxxxxxxxxxxxxxxxxxxxxxxxxxxxxxxxxxxxxxxxxxxxxxxxxxxxxxxxxxxxxxxxxxx
