# Supplementary material for: Mothers’ satisfaction with health extension services and the associated factors in Gamo Goffa zone, Southern Ethiopia
Source: PLoS One. 2020 May 7;15(5):e0232908. doi: 10.1371/journal.pone.0232908 (PMC7205287; doi:10.1371/journal.pone.0232908)
Supplement: Attachment — Submitted filename: Response to Reviewers.docx [file pone.0232908.s004.docx]

Authors’ response to Editor and Reviewer comments

Additional Editor Comments (if provided):

Comment:

Dear Author/ Authors,
The opinions and suggestions of the 3rd reviewer regarding your article are below. Submit corrections in your article according to the recommendations of the reviewer.

Response: Thank you dear Prof. Nülüfer Erbil, we have given a point-by-point response to the reviewer’s comment and have made the changes in the revised manuscript shown with track changes.
xxxxxxxxxxxxxxxxxxxxxxxxxxxxxxxxxxxxxxxxxxxxxxxxxxxxxxxxxxxxxxxxxxxxxxxxxxxxxxxxxxxxxxxxxxxxxxxxxxxxxxxxxxxxxxxxxxxxxxxxxxxxxxxxxxxxxxxxxxxxxxxxxxxxxxxxxxxx

**Reviewer 3 comments**
**General comments:** Thank you for follow my recommendations.

Response: Thank you for your inspiring comment.

**Specific comments:**

Please see care full until now some comment not corrected like the following.
1- It's better to capitalize the first letter in each word in the title.

Response: As we have replied in the first revision, we left it as it is just to conform to Plos One title guide which recommends the title to be in a sentence case. Kindly consider our efforts to conform to the journal formatting style. (Please refer to <https://journals.plos.org/plosone/s/submission-guidelines>, specifically under the section of **Title page**
2- At the beginning of each paragraph leaves a space and begins writing.

Response: We ask apologies for missing this comment. We have now corrected this in the revised submission.

3- Some of grammatical errors should be corrected

Response: We are so sorry; we could not get the exact places in the document where these corrections are actually needed. However, we have gone all over the texts and corrected the errors we are able to detect.
4- in line 41 you are corrected the style of writing. For example, the [AOR=4.02 (95% CI 45 2.0, 8.1), You are corrected it [AOR=4.02 (95% CI 45 2.0, 8.1)],
Correct all of them in line 42, 43,44…. When you used the [ at the begin should be closed at the end. [……] . If I was right.

Response: Thank you very much for your meticulous observation to the errors we were unable to detect. We have corrected it and have made the changes in the “revised man­uscript with track changes” file.
5- Number and data of Ethics consideration letter write down under subtitle Ethics consideration

Response: We have included the number and date from the ethics assurance letter under the “Ethics consideration” section of methods.

6- Table number 2, unifying the form of writing numbers and the symbol %
For example, you are put in some area or part the symbol of percentage and omitted in the other part.

Response: We have corrected in accordance with your recommendations in Table 2.

xxxxxxxxxxxxxxxxxxxxxxxxxxxxxxxxxxxxxxxxxxxxxxxxxxxxxxxxxxxxxxxxxxxxxxxxxxxxxxxxxxxxxxxxxxxxxxxxxxxxxxxxxxxxxxxxxxxxxxxxxxxxxxxxxxxxxxxxxxxxxxxxxxxxxxxxxxxx
